# Tumor burden score and carcinoembryonic antigen predict outcomes in patients with intrahepatic cholangiocarcinoma following liver resection: a multi‑institutional analysis

**DOI:** 10.1186/s12885-024-12091-2

**Published:** 2024-03-20

**Authors:** Jun Fu, Lifang Zheng, Shicuan Tang, Kongying Lin, Shuguo Zheng, Xinyu Bi, Jianming Wang, Wei Guo, Fuyu Li, Jian Wang, Kui Wang, Haitao Li, Yongyi Zeng

**Affiliations:** 1 Department of Hepatobiliary Surgery, Mengchao Hepatobiliary Hospital of Fujian Medical University, 312 Xihong Road, Fuzhou, China; 2 Department of Hepatopancreatobiliary Surgery, First Affiliated Hospital of Fujian Medical University, Fuzhou, China; 3 Department of Hepatobiliary Surgery, The Southwest Hospital Affiliated to the Army Medical University, Chongqing, China; 4 Department of Hepatobiliary Surgery, Cancer Hospital, Chinese Academy of Medical Sciences, Beijing, China; 5 Department of Hepatobiliary Surgery, Tongji Hospital Affiliated to Tongji Medical College, Huazhong University of Science Technology, Wuhan, China; 6 Department of Hepatobiliary Surgery, Beijing Friendship Hospital Affiliated to Capital Medical University, Beijing, China; 7 Department of Hepatobiliary Surgery, The West China Hospital of Sichuan University, Chengdu, China; 8 Department of Hepatobiliary Surgery, Renji Hospital, Shanghai Jiaotong University, Shanghai, China; 9 Department of Hepatic Surgery (II), Eastern Hepatobiliary Surgery Hospital, Navy edical University, Shanghai, China

**Keywords:** Primary liver cancer, Tumor burden score, CEA, Multi − institutional database, Prediction model

## Abstract

**Background:**

The prognostic significance of tumor burden score (TBS) in relation to carcinoembryonic antigen (CEA) has not been investigated among patients undergoing hepatectomy for intrahepatic cholangiocarcinoma (ICC). This study aimed to develop and validate a simplified model, a combination of TBS and CEA (CTC grade), for predicting the long-term outcomes of postoperative ICC patients.

**Methods:**

Patients who underwent curative − intent resection of ICC between 2011 and 2019 were identified from a large multi − institutional database. The impact of TBS, CEA, and the CTC grade on overall survival (OS) and recurrence − free survival (RFS) was evaluated in both the derivation and validation cohorts. The receiver operating characteristic curve was utilized for assessing the predictive accuracy of the model. Subgroup analyses were performed across 8th TNM stage system stratified by CTC grade to assess the discriminatory capacity within the same TNM stage.

**Results:**

A total of 812 patients were included in the derivation cohort and 266 patients in the validation cohort. Survival varied based on CEA (low: 36.7% vs. high: 9.0%) and TBS (low: 40.3% vs. high: 17.6%) in relation to 5 − year survival (both *p* < 0.001). As expected, patients with low CTC grade (i.e., low TBS/low CEA) were associated with the best OS as well as RFS, while high CTC grade (i.e., high TBS/high CEA) correlated to the worst outcomes. The model exhibited well performance in both the derivation cohort (area under the curve of 0.694) and the validation cohort (0.664). The predictive efficacy of the CTC grade system remains consistently stable across TNM stages I and III/IV.

**Conclusion:**

The CTC grade, a composite parameter derived from the combination of TBS and CEA levels, served as an easy − to − use tool and performed well in stratifying patients with ICC relative to OS and RFS.

**Supplementary Information:**

The online version contains supplementary material available at 10.1186/s12885-024-12091-2.

## Introduction

Intrahepatic cholangiocarcinoma (ICC), a rare malignant tumor originating from the epithelium lining and peribiliary glands of the secondary bile ducts extending to the terminal branches of the intrahepatic biliary tree, has seen an increasing incidence in recent decade and now accounting for approximately 10 − 15% of primary liver cancer (PLC) [1, 2]. Surgical resection remains the fundamental and potentially the most efficacious approach for managing patients with resectable ICC [3]. Unfortunately, only a small subset of patients with ICC present with resectable tumors, and long − term outcomes after even curative − intent resection remain dismal, with reported 5-year overall survival rates ranging from 20–35% [4]. This can be largely attributed to the tumor progression and the nature history of recurrence [5]. Even among patients with similar tumor morphology characteristics (e.g., tumor size and tumor number) and comparable tumor biological markers (e.g., carcinoembryonic antigen, abbreviated as CEA) or within the same TNM stage, there remains significant diversity in outcomes following ICC resection [6, 7]. Given the suboptimal outcomes frequently observed postoperatively, there has been intense interest in identifying means to select patients better in the preoperative setting who might benefit more from curative − intent resection of ICC and who are better candidates for neoadjuvant treatment strategies.

To date, only a few clinical staging systems and personalized prediction models for ICC have been established to better understand the prognosis of specific patient groups and provide individual prognostic predictions [8–12]. For instance, the Tumor − Node − Metastasis (TNM) classification system of ICC, promulgated by the American Joint Committee on Cancer (AJCC) and the Union for International Cancer Control, is the most extensively employed and accepted staging schema in clinical practice [8]. In addition, the utilization of an alternative Liver Cancer Study Group of Japan (LCSGJ) staging system is infrequent and limited to local practices [11]. However, the common predictors of these staging systems, such as tumor size, tumor number, vascular invasion, lymph node metastasis, and extrahepatic metastasis, are solely based on post − operative histopathological examination and cannot be effectively assessed preoperatively. Simultaneously, Wang et al. [12] and Hyder et al. [9] have sequentially established individual prediction models using nomograms that incorporate numerous clinical and pathological indicators. Despite the favorable predictive performance demonstrated by these models, the complexity of these prognostic models restricts their practicality and widespread application in patient care, even after validation [13]. Establishing a preoperative prediction model with minimal parameters is urgently required to provide comprehensive prognostic information, accurately assess which patients may benefit the most from a given treatment, and inform discussions on long-term outcomes [14].

The TBS, initially proposed by Sasaki et al. [15], is a comprehensive morphological measure that integrates tumor size and tumor number for patients undergoing hepatic resection of colorectal liver metastasis (CRLM). Traditional assessments of tumor burden typically involve the tumor size and tumor number, with both variables having been incorporated into the AJCC − ICC − TNM staging system [8]. However, in the majority of prognostic prediction models, when shifting these two continuous variables to categorical variables, the selection of cutoff values is often relatively arbitrary [16–18]. TBS overcomes the limitations of this category classification by utilizing continuous variables, thereby avoiding arbitrary cutoff values that may diminish statistical power and potentially result in inaccurate inferences [19]. In recent years, there has been a growing interest in studies on TBS in PLC, making it a clinical research hotspot. The TBS model has demonstrated its prognostic risk stratification capabilities in hepatocellular carcinoma (HCC) [20], ICC [21], and combined hepatocellular − cholangiocarcinoma [22], while remaining user − friendly. Relevant studies have shown a strong correlation between radiological TBS and pathological TBS, with no significant difference observed in postoperative prediction ability [23, 24]. Furthermore, several studies have suggested the integration of TBS with tumor markers (e.g., AFP, CA19 − 9) [25, 26] or liver function surrogate index (e.g., ALBI) [27] to develop innovative classification models. These prognostic models have demonstrated effectiveness in prognostic stratification, surpassing the standalone TBS model in certain scenarios.

Elevations in tumor markers, such as CEA, also serves as a significant adverse prognostic factor for ICC, indicating unfavorable tumor biology [28, 29]. In 2022, Sanchez et al. [7] demonstrated elevated serum CEA levels (cutoff value, 5 IU/ml) was observed in patients with locally advanced or metastatic (both *p* < 0.001) ICC when compared to those with earlier − stage, liver − confined disease. Recently, Moazzam et al. [30] utilized international multi − institutional data to incorporate TBS and CA19 − 9 into a composite CTC grading system, allowing for the categorization of patients into distinct subgroups. Importantly, higher CTC grades were correlated with worse outcomes in terms of recurrent free survival (RFS) and overall survival (OS). However, it is worth noting that most of these studies lack validation cohorts, which somewhat limiting their generalizability.

To date, there is a lack of literature reporting the efficacy of models that combine radiological TBS and serum CEA levels in preoperative evaluation. The objective of this study was to develop and externally validate a simplified prognostic CTC grading system through a large sample, multi − institutional database that is user − friendly in clinical practice, applicable in a preoperative setting, highly accurate, and discriminatory.

## Methods

### Study population and selection criteria

A retrospective study was conducted, collecting data from eight large tertiary medical institutions in China from 2011 to 2019. The derivation cohort data (812 patients) were collected from the Primary Liver Cancer Big Data (PLCBD) system [31], which includes Mengchao Hepatobiliary Hospital of Fujian Medical University and Eastern Hepatobiliary Surgery Hospital of Naval Medical University. The data from the PLCBD system are prospectively collected and updated annually since 2019. The validation cohort data (266 patients) were obtained from the electronic medical record systems of The Southwest Hospital Affiliated to the Army Medical University, Beijing Friendship Hospital Affiliated to Capital Medical University in Beijing, Cancer Hospital of Chinese Academy of Medical Sciences, Tongji Hospital Affiliated to Tongji Medical College, Renji Hospital Affiliated to Shanghai Jiaotong University, and West China Hospital of Sichuan University. The patients in this study all provided informed consent prior to surgery, and strict adherence was maintained to the guidelines of the Declaration of Helsinki. Ethical approval was obtained from the Institutional Review Boards of all participating institutions (approval number 2022_077_01).

The inclusion criteria were as follows: (1) ECOG score of 0 − 2, (2) Child–Pugh score from A5 to B7, (3) Dynamic contrast − enhanced computed tomography (CT) or magnetic resonance imaging (MRI) assessment of tumor size and number within one month before surgery, (4) serum CEA level measured within one week before surgery, (5) undergoing curative − intent resection, (6) with postoperative pathological confirmation of ICC. The exclusion criteria were as follows: (1) Postoperative pathological confirmation of cHCC − CCA, (2) R2 resection, (3) receipt of therapies such as radiofrequency ablation, local interventional procedures, or chemotherapy before surgery, (4) recurrent ICC or simultaneously having malignancies in other anatomical sites, (5) Patients who died within 30 days postoperatively or were lost to follow − up shortly after surgery. Unexpectedly, intraoperative identification of extra − regional lymph node spread, peritoneal metastasis, and mesenteric metastasis, requiring concurrent resection with the primary lesion for complete eradication, and these data were not excluded.

### Variables of interested

Demographic and clinicopathologic data were collected, including age, gender, hepatitis B virus (HBV) infection, American Society of Anesthesiologists Physical Status Classification (ASA) score, presence of cirrhosis, Child − Pugh grade. Hematological parameters included platelet count, neutrophil count, lymphocyte count, as well as CA19 − 9 and CEA levels. Neutrophil count and lymphocyte count together constituted the Neutrophil − to − Lymphocyte Ratio (NLR). Tumor number and the size of the largest lesion were evaluated by CT or MRI. Furthermore, data on the type of resection, histologic tumor grade, presence of macrovascular invasion, microvascular invasion, margin status [i.e., microscopically negative (R0), positive (R1)], postoperative 30 − day complication, and receipt of adjuvant therapy were also recorded. The TNM staging was performed according to the 8th edition of the AJCC staging manual.

### TBS definition and CTC Grade evaluation

TBS is calculated as the Euclidean distance in a Cartesian plane, considering two variables: maximum tumor size (x − axis) and the number of tumors (y − axis). For patients with multiple nodules, tumor size was defined as the size of the largest lesion. The formula for TBS computation follows the Pythagorean theorem: TBS^2^ = (the size of the largest lesion)^2^ + (number of tumors)^2^.

The receiver operating characteristic (ROC) analysis was utilized to determine the optimal cutoff values for TBS (6.1 units) and CEA (5.1 U/mL). TBS and CEA levels were then classified into low and high categories, respectively. Subsequently, CTC grades were established by combining the categories: low CTC grade (low TBS/low CEA), intermediate CTC grade (low TBS/high CEA or high TBS/low CEA), and high CTC grade (high TBS/high CEA).

### Definition of other important clinical and follow − up related variables

Major hepatectomy was defined as resection of three or more Couinaud segments according to the consensus of the Brisbane 2000 system [32]. Postoperative complications occurring within 30 days were classified according to the Clavien − Dindo classification [33]. The curative − intend surgery was to completely remove the macroscopic tumors with adequate resection margins. The presence of visible tumor remnants at the surgical margin was classified as an R2 resection and excluded from the study. Microvascular invasion (MVI) was defined as the presence of intraparenchymal vascular involvement identified on histological examination. Macrovascular invasion was defined as the involvement of primary and secondary branches of the portal vein or hepatic artery, or the invasion of one or more of the three major hepatic veins [8]. The primary outcome of interest was OS, defined as the time interval between the date of ICC liver resection and the date of death or last follow − up. Secondary outcome was RFS, defined as the time between resection of ICC and date of recurrence or last follow − up. Recurrence of ICC was confirmed either by tumor biopsy or the identification of a suspicious lesion on follow − up imaging.

### Statistical analysis

Continuous variables were presented as mean ± standard deviation (SD) or as median and interquartile range (IQR). Differences were assessed using one-way analysis of variance (ANOVA) or the Kruskal-Wallis H tests (K-W tests). Categorical variables were expressed as frequencies and percentages, and group comparisons were conducted using the Chi-square test. Survival analyses for OS and RFS were performed through Kaplan-Meier (KM) curves, with differences evaluated by the log-rank test. Independent prognostic factors for OS and RFS were identified using Cox regression analyses. The optimal cutoff values for TBS and CEA were determined through ROC analysis by maximizing the Youden Index. The discriminative ability of the model was analyzed by the Harrell c-index and areas under the receiver operating characteristic curve (AUC). Calibration of the model was analyzed using the calibration plot with Hosmer-Lemeshow-Test. Statistical analysis was executed using SPSS® version 25.0 (IBM, Armonk, New York, USA) and R program version 3.2.0 (http://www.r-project.org/). A *p*-value of less than 0.05 was considered statistically significant.

## Results

### Baseline characteristics of derivation and validation cohorts

A total of 1078 ICC patients include 812 patients in the derivation cohort and 266 patients in the validation cohort were retrospectively reviewed. As summarized in Table 1 and Supplement Table 1, mean age were 54.0 years and 54.1 years, with the majority of patients being male in the derivation (*n* = 554, 68.2%) and validation (*n* = 170, 63.9%) cohorts. In the derivation cohort, the proportion of patients with HBV infection (*n* = 310, 38.1% vs. *n* = 125, 46.9%) was lower than that in the validation cohort, while the proportion of patients with liver cirrhosis (*n* = 343, 42.2% vs. *n* = 81, 30.4%) was higher. The mean and standard deviation of maximum tumor size were 6.3 ± 3.5 cm and 6.5 ± 3.1 cm, respectively. Multiple tumors were present in only 10.0% (*n* = 82) of patients in the derivation cohort while 15.7% (*n* = 42) of patients in the validation cohort. In both groups, 315 (38.7%) patients and 62 (23.3%) patients, respectively, underwent major hepatectomy. Majority of patient received R0 resection (*n* = 733, 90.3% vs. *n* = 235, 88.4%). The proportion of postoperative 30 − day complications (*n* = 343, 42.2% vs. *n* = 99, 37.2%) were slightly higher in the derivation cohort compared to the validation cohort.

**Table 1:** Demographic and clinical characteristics of patients in the derivation cohort

N (%)	All (*n* = 812, 100%)	Low CTC (*n* = 332, 39.7%)	Intermediate CTC (*n* = 354, 43.6%)	High CTC (*n* = 126, 15.5%)	*P* value
Age, years*	54.0 ± 10.7	53.8 ± 10.5	54.7 ± 10.9	55.1 ± 10.6	0.084
Gender, Male	554 (68.2)	232 (69.8)	242 (68.3)	80 (63.4)	0.422
HBV (+)	310 (38.1)	134 (40.3)	139 (39.2)	37 (29.3)	0.082
ASA score ≥ 2	187 (23.0)	82 (24.6)	77 (21.7)	28 (22.2)	0.639
Cirrhosis	343 (42.2)	154 (46.3)	143 (40.3)	46 (36.5)	0.104
Child-Pugh grade B	44 (5.4)	18 (5.4)	20 (5.6)	6 (4.7)	0.931
Preoperative platelet counts, ×10^9^/L*	192.6 ± 72.6	179.7 ± 74.0	198.6 ± 71.9	210.1 ± 65.2	**< 0.001**
Preoperative NLR*	2.5 (1.9–3.6)	2.1 (1.6–2.8)	2.9 (2.1–4.1)	3.0 (2.2–4.1)	**< 0.001**
Preoperative CA 19 − 9, U/mL*	39.5 (15.7–247.4)	28.8 (14.7–94.6)	41.2 (14.4–236.1)	378.9 (25.7–1000.0)	**< 0.001**
Preoperative CEA (ng/mL), n(%)					**< 0.001**
Low	603 (74.3)	332 (100.0)	271 (76.5)	0 (0.0)	
High	209 (25.7)	0 (0.0)	83 (23.5)	126 (100.0)	
Tumor Burden Score					**< 0.001**
Low	415 (51.1)	332 (100.0)	83 (23.5)	0 (0.0)	
High	397 (48.9)	0 (0.0)	271 (76.5)	126 (100.0)	
Maximum tumor size, cm*	6.3 ± 3.5	3.7 ± 1.2	7.9 ± 3.9	8.4 ± 2.0	**< 0.001**
Tumor number, multiple	82 (10.0)	30 (9.0)	37 (10.4)	15 (11.9)	0.633
Tumor differentiation					0.102
Well	78 (9.6)	32 (9.6)	40 (11.3)	6 (4.8)	
Poor to moderate	734 (90.4)	300 (90.4)	314 (88.7)	120 (95.2)	
Type of resection, Major	315 (38.7)	97 (29.2)	154 (43.5)	64 (50.7)	**< 0.001**
Macrovascular invasion	107 (13.1)	30 (9.0)	55 (15.5)	22 (17.4)	**0.013**
Microvascular invasion	125 (15.3)	46 (13.8)	65 (18.3)	14 (11.1)	0.092
Resection margin status					**0.011**
R0	733 (90.3)	312 (94.0)	309 (87.3)	112 (88.8)	
R1	79 (9.7)	20 (6.0)	45 (12.7)	14 (11.2)	
Postoperative adjuvant therapy	255 (31.4)	100 (30.1)	119 (33.6)	36 (28.5)	0.466
AJCC staging system^8th^					**0.007**
I	437 (53.8)	204 (61.4)	175 (49.4)	58 (46.0)	
II	163 (20.1)	63 (19.0)	74 (20.9)	26 (20.6)	
III	189 (23.3)	60 (18.1)	91 (25.8)	38 (30.2)	
IV	23 (2.8)	5 (1.5)	14 (3.9)	4 (3.2)	
Postoperative 30-day complication	343 (42.2)	128 (38.5)	157 (44.3)	58 (46.0)	0.198
Minor morbidity	274 (33.7)	104 (31.3)	122 (34.4)	48 (38.1)	0.365
Major morbidity	69 (8.5)	24 (7.2)	35 (9.9)	10 (7.9)	0.445

ASA, American Society of Anesthesiologists; AJCC, American Joint Committee on Cancer; CA 19 − 9, carbohydrate antigen 19 − 9; CEA, carcinoembryonic antigen; CTC, combination of Tumor Burden Score and CEA; HBV, hepatitis B virus; NLR, neutrophil-to-lymphocyte ratio

* Values are mean ± standard deviation or median (interquartile range) unless otherwise indicated

CEA levels were dichotomized into high and low grades using a cutoff value of 5.1 U/ml. In the derivation cohort, 603 (74.3%) patients were classified into the low CEA grade, while 209 (25.7%) patients were in the high CEA grade. In the validation cohort, 208 (78.2%) patients were classified into the low CEA grade, and 58 (21.8%) patients were in the high CEA grade. Similarly, TBS was divided into high and low TBS grade using a cutoff value of 6.1 unit. In the derivation cohort, 415 (51.1%) patients were in the low TBS grade, and 397 (48.9%) were in the high TBS grade. In the validation cohort, 123 (46.2%) patients were in the low TBS grade, and 143 (53.8%) were in the high TBS grade. According to the 8th edition AJCC classification, approximately half of the patients were in the stage I (*n* = 437, 53.8%, in the derivation cohort, vs. *n* = 128, 48.1%, in the validation cohort).

### Association between CTC grade and clinicopathologic features

The CTC grades were determined based on the following categories: low CTC grade (low TBS/low CEA), intermediate CTC grade (low TBS/high CEA or high TBS/low CEA), and high CTC grade (high TBS/high CEA). In the derivation cohort, preoperative platelet counts (mean ± standard deviation, ×109/L; low CTC grade, 179.7 ± 74.0 vs. intermediate CTC grade, 198.6 ± 71.9 vs. high CTC grade, 210.1 ± 65.2, respectively, *P*< 0.001), NLR [median and interquartile range; low CTC grade, 2.1 (1.6 − 2.8) vs. intermediate CTC grade, 2.9 (2.1 − 4.1) vs. high CTC grade, 3.0 (2.2 − 4.1), respectively, *P*< 0.001], and CA 19 − 9 level [U/mL, median and interquartile range; low CTC grade, 28.8 (14.7 − 94.6) vs. intermediate CTC grade, 41.2 (14.4 − 236.) vs. high CTC grade, 378.9 (25.7 − 1000.0), respectively, *p* < 0.001] increased incrementally with the CTC grade. Additionally, the proportions of major hepatectomy (low CTC grade, *n* = 97, 29.2% vs. intermediate CTC grade, *n* = 154, 43.5%, high CTC grade, *n* = 64, 50.7%, respectively, *P*< 0.001) and macrovascular invasion (low CTC grade, *n* = 30, 9.0% vs. intermediate CTC grade, *n* = 55, 15.5%, high CTC grade, *n* = 22, 17.4%, respectively, *p* < 0.001) increased gradually with higher CTC grade. However, there were no differences in postoperative adjuvant therapy and postoperative 30 − day complications among CTC grade (Table 1). Similar results were observed in the validation group (supplement Table 1). The presence of a higher CTC grade indicates a larger proportion of a AJCC stage II and higher stage patients (low CTC grade, *n* = 128, 38.6% vs. intermediate CTC grade, *n* = 179, 50.6%, high CTC grade, *n* = 68, 54%, respectively, *p* = 0.007).

### Related factors affecting OS and RFS in the derivation and validation cohorts

In the multivariate Cox model in the derivation cohort, only CA19 − 9 > 37 U/mL [Hazard Ratio (HR): 1.42, 95% CI: 1.20–1.67, *p* < 0.001], the 8th AJCC staging system (HR: 1.96, 95% CI: 1.67–2.40, *p* < 0.001), and higher CTC grade (Intermediate vs. Low, HR: 2.06, 95% CI: 1.59–2.51; High vs. Low, HR: 3.34, 95% CI: 2.58–4.35; both *p* < 0.001) were independent predictors of increased mortality (Table 2). In the multivariate Cox model for RFS, ASA score ≥ 2 (HR: 1.36, 95% CI: 1.13–1.66, *p* < 0.001), CA19 − 9 > 37 U/mL (HR: 1.25, 95% CI: 1.09–1.57, *p* = 0.004), the 8th AJCC staging system (HR: 1.72, 95% CI: 1.42–2.08, *p* < 0.001), and higher CTC grade (Intermediate vs. Low, HR: 1.66, 95% CI: 1.35–2.05; High vs. Low, HR: 2.55, 95% CI: 1.97–3.24; both *p* < 0.001) were independent predictors of increased recurrence (Table 3). In the multivariate Cox model in the validation cohort, an ASA score ≥ 2 was not an independent risk factor for recurrence. Besides CA19 − 9 > 37 U/mL, the 8th AJCC staging system, and higher CTC grade, macrovascular invasion and microvascular invasion were also identified as independent predictors for both OS and RFS (Supplement Table 2 and Supplement Table 3).

**Table 2:** Univariable and multivariable Cox-regression analysis for overall survival (OS) of patients in the derivation cohort

Variables	HR comparison	UV HR (95% CI)	UV *P* value	MV HR (95% CI)	MV *P* value	
Age	> 60 vs. ≤ 60 years	1.21 (1.00–1.46)	**0.049**	NS	0.058	
Gender	Male vs. Female	0.91 (0.75–1.10)	0.354			
HBV (+)	HBV vs. non-HBV	0.87 (0.72–1.05)	0.151			
ASA score	≥ 2 vs. < 2	1.20 (0.98–1.47)	0.067			
Cirrhosis	Yes vs. No	1.26 (1.05–1.65)	**0.011**	NS	0.082	
Child-Pugh grade	B vs. A	1.31 (0.88–1.93)	0.171			
Preoperative platelet counts	< 100 vs. ≥ 100 × 10^9^/L	0.70 (0.50–1.09)	0.063			
Preoperative NLR	> 4 vs. ≤ 4	1.52 (1.23–1.88)	**< 0.001**	NS	0.165	
Preoperative CA 19 − 9	> 37 vs. ≤ 37 U/mL	1.83 (1.53–2.20)	**< 0.001**	1.42 (1.20–1.67)	**< 0.001**	
Tumor differentiation	Poor or moderate vs. Well	1.03 (0.97–1.38)	0.057			
Type of resection	Major vs. Minor	1.36 (1.13–1.62)	**< 0.001**	NS	0.802	
Macrovascular invasion	Yes vs. No	1.37 (1.07–1.76)	**0.012**	NS	0.521	
Microvascular invasion	Yes vs. No	1.34 (1.06–1.69)	**0.011**	NS	0.232	
Resection margin status	R1 vs. R0	1.69 (1.35–1.93)	**0.015**	NS	0.874	
Postoperative adjuvant therapy	Yes vs. No	0.92 (0.76–1.11)	0.404			
AJCC staging system^8th^	III/IV vs. I/II	2.34 (1.93–2.83)	**< 0.001**	1.96 (1.61–2.40)	**< 0.001**	
CTC grade	Intermediate vs. Low	1.82 (1.48–2.24)	**< 0.001**	2.06 (1.59–2.51)	**< 0.001**	
	High vs. Low	2.62 (2.00–3.43)	**< 0.001**	3.34 (2.58–4.35)	**< 0.001**	

ASA, American Society of Anesthesiologists; AJCC, American Joint Committee on Cancer; CA 19 − 9, carbohydrate antigen 19 − 9; CEA, carcinoembryonic antigen; CTC, combination of Tumor Burden Score and CEA; CI, confidence interval; HBV, hepatitis B virus; HR, hazard ratio; MV, multivariable; NLR, neutrophil-to-lymphocyte ratio; NS, not significant; UV, univariable

**Table 3:** Univariable and multivariable Cox-regression analysis for recurrence-free survival (RFS) of patients in the derivation cohort

Variables	HR comparison	UV HR (95% CI)	UV *P* value	MV HR (95% CI)	MV *P* value
Age	> 60 vs. ≤ 60 years	1.18 (0.93–1.34)	0.229		
Gender	Male vs. Female	0.93 (0.78–1.11)	0.455		
HBV (+)	HBV vs. non-HBV	1.00 (0.85–1.19)	0.924		
ASA score	≥ 2 vs. < 2	1.34 (1.11–1.61)	**0.002**	1.36 (1.13–1.67)	**0.002**
Cirrhosis	Yes vs. No	1.31 (1.09–1.57)	**0.021**	NS	0.087
Child-Pugh grade	B vs. A	1.06 (0.72–1.55)	0.757		
Preoperative platelet counts	< 100 vs. ≥ 100 × 10^9^/L	0.73 (0.53 -1.00)	0.051		
Preoperative NLR	> 4 vs. ≤ 4	1.39 (1.14–1.70)	**0.001**	NS	0.614
Preoperative CA 19 − 9	> 37 vs. ≤ 37 U/mL	1.55 (1.31–1.83)	**< 0.001**	1.25 (1.09–1.57)	**0.007**
Tumor differentiation	Poor or moderate vs. Well	1.02 (0.74–1.31)	0.918		
Type of resection	Major vs. Minor	1.23 (1.03–1.45)	**0.016**	NS	0.683
Macrovascular invasion	Yes vs. No	1.37 (1.08–1.74)	**0.009**	NS	0.096
Microvascular invasion	Yes vs. No	1.35 (1.09–1.68)	**0.006**	NS	0.172
Resection margin status	R1 vs. R0	1.31 (1.01–1.72)	**0.044**	NS	0.412
Postoperative adjuvant therapy	Yes vs. No	0.99 (0.83–1.18)	0.927		
AJCC staging system^8th^	III/IV vs. I/II	2.34 (1.93–2.83)	**< 0.001**	1.72 (1.42–2.08)	**< 0.001**
CTC grade	Intermediate vs. Low	1.79 (1.48–2.16)	**< 0.001**	1.66 (1.35–2.05)	**< 0.001**
	High vs. Low	2.75 (2.17–3.49)	**< 0.001**	2.55 (1.97–3.24)	**< 0.001**

ASA, American Society of Anesthesiologists; AJCC, American Joint Committee on Cancer; CA 19 − 9, carbohydrate antigen 19 − 9; CEA, carcinoembryonic antigen; CTC, combination of Tumor Burden Score and CEA; CI, confidence interval; HBV, hepatitis B virus; HR, hazard ratio; MV, multivariable; NLR, neutrophil-to-lymphocyte ratio; NS, not significant; UV, univariable

### Effects of TBS, CEA, and CTC on OS and RFS

**Fig. 1:**
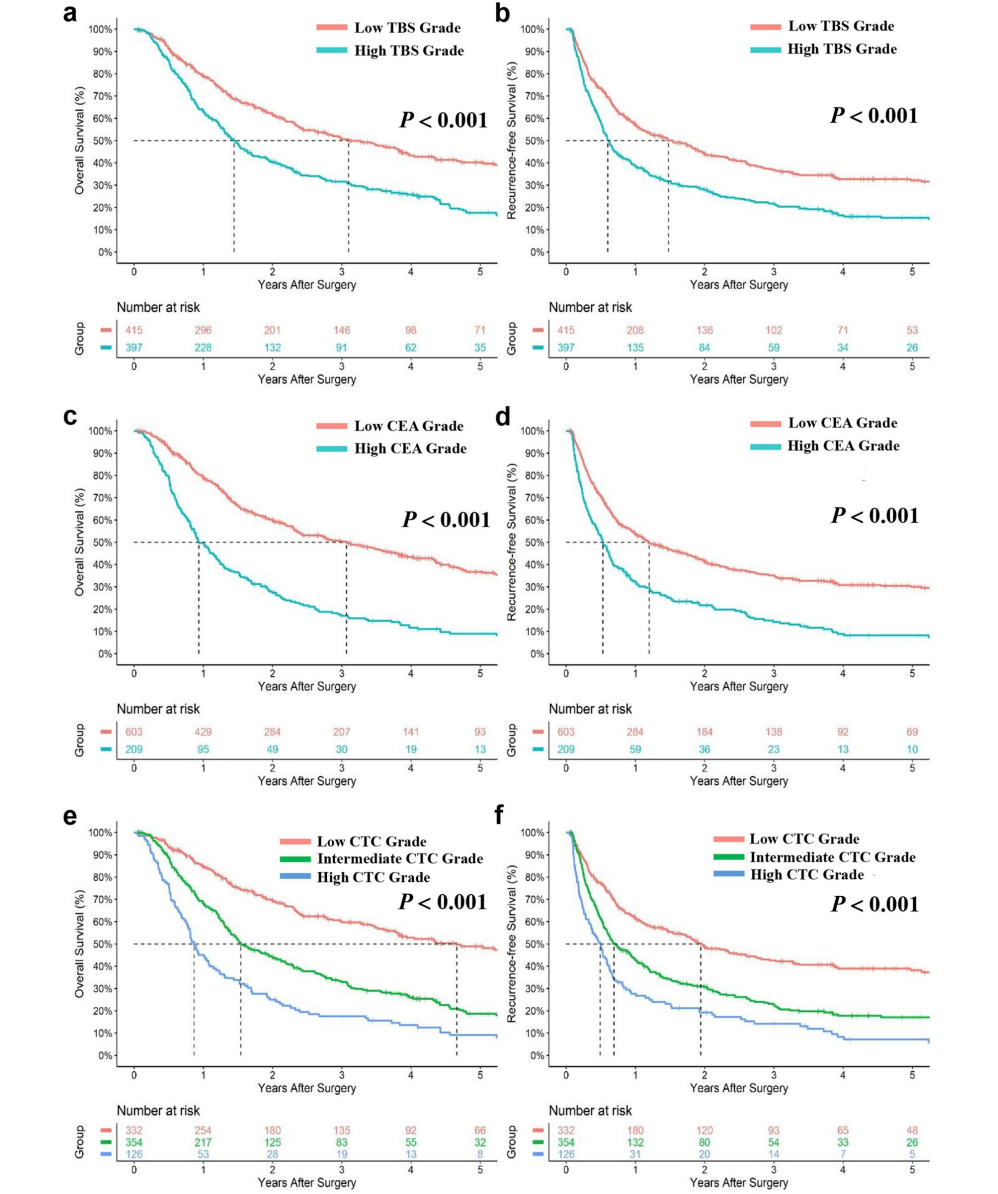
Cumulative overall survival (OS) and recurrence-free survival (RFS) curves of patients stratified by TBS (**a, b**), CEA (**c, d**), and CTC grade (**e, f**) in the derivation cohort. CEA, carcinoembryonic antigen; CTC, combination of Tumor Burden Score and CEA; TBS, Tumor Burden Score

**Fig. 2:**
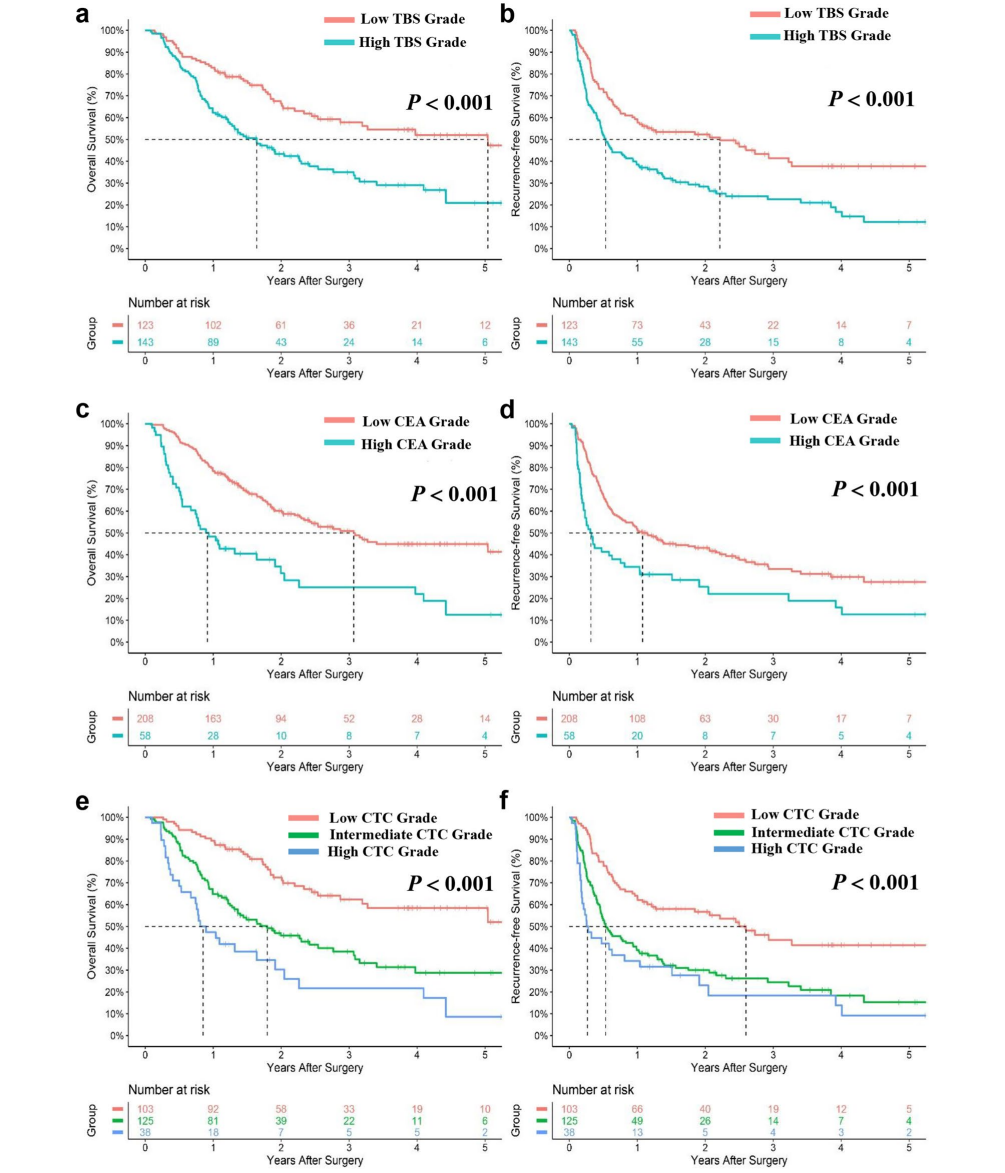
Cumulative overall survival (OS) and recurrence-free survival (RFS) curves of patients stratified by TBS (**a, b**), CEA (**c, d**), and CTC grade (**e, f**) in the validation cohort. CEA, carcinoembryonic antigen; CTC, combination of Tumor Burden Score and CEA; TBS, Tumor Burden Score

At the end of the last follow − up, a total of 452 (56.7%) patients died and 480 (59.1%) patients recured, with the median OS and RFS were 33.0 months and 19.6 months, respectively, in the derivation cohort. Patients with high TBS grade had markedly worse 5 − year OS and RFS compared with patients with low TBS grade (5 − year OS: 17.6% vs. 40.3%, 5 − year RFS: 15.4% vs. 32.1%, respectively, both *p* < 0.001) (Fig. 1a and b); similarly, patients with high CEA level had markedly worse 5 − year OS and RFS compared with low CEA level (5 − year OS: 9.0% vs. 36.7%, 5 − year RFS: 8.2% vs. 30.0%, respectively, both *p* < 0.001) (Fig. 1c and d). Higher CTC grades had an incremental worse OS (1−, 3 − 5−year OS: low CTC, 84.8%, 59.7%, 48.9% vs. intermediate CTC, 68.3%, 33.0%, 18.7% vs. high CTC, 45.1%, 17.6%, 9.1%; respectively, *p* < 0.001) and RFS (1−, 3 − 5−year RFS: low CTC, 61.8%, 42.6%, 38.2% vs. intermediate CTC, 43.0%, 22.8%, 17.1% vs. high CTC, 27.6%, 14.2%, 7.2%; respectively, *p* < 0.001) (Fig. 1e and f). In the validation cohort, Kaplan − Meier survival curves demonstrated significant differentiation in each group, with all *p* < 0.001 (Fig. 2).

**Fig. 3:**
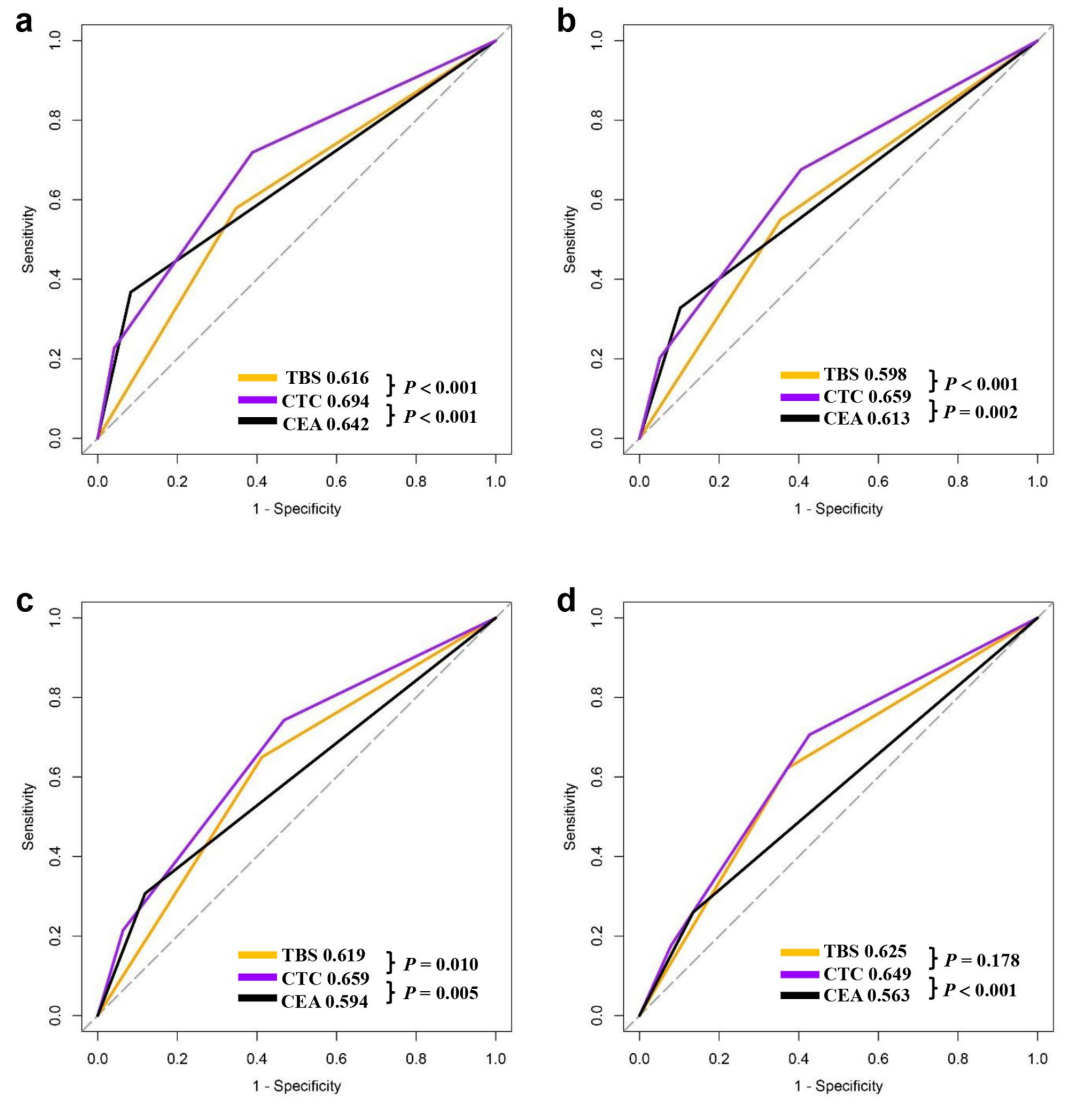
Comparison of the predictive value of TBS, CEA, and CTC grade in overall survival (OS) (**a** and **c**) and recurrence-free survival (RFS) (**b** and **d**) in the derivation cohort and validation cohort. CEA, carcinoembryonic antigen; CTC, combination of Tumor Burden Score and CEA; TBS, Tumor Burden Score

The ROC curve of CTC grading system showed an AUC value of 0.694 (95% CI = 0.661–0.727) for OS and 0.659 (95% CI = 0.623–0.694) for RFS in the derivation cohort (Fig. 3a and b). These results indicated that the CTC grading system exhibited a moderate to well prognosis predictive performance, surpassed those of individual TBS (OS: AUC 0.616, 95% CI = 0.581–0.649, *p* < 0.001; RFS: 0.598, 95% CI = 0.562–0.634, *p* < 0.001) and CEA level (OS: AUC 0.642, 95% CI = 0.616–0.727, *p* < 0.001; RFS: 0.613, 95% CI = 0.586–0.639, *p* = 0.002) assessments. In the validation cohort, CTC grading system demonstrated moderate to well prognostic prediction capabilities (OS: AUC 0.664, 95% CI = 0.605–0.723, *p* < 0.001; RFS: 0.649, 95% CI = 0.585–0.713, *p* < 0.001), outperforming both TBS (AUC 0.619, 95% CI = 0.560–0.677, *p* = 0.010) and CEA levels (AUC 0.594, 95% CI = 0.546–0.641, *p* = 0.005) for predicting OS (Fig. 3c and d). For calibration of the CTC model, calibration plots depicted a good consistency between the predicted outcome and the observed outcome of the model in terms of 5-year OS and RFS in the derivation and validation cohorts (supplemented Fig. 1).

### CTC grading system predicts OS and RFS across AJCC stages

**Fig. 4:**
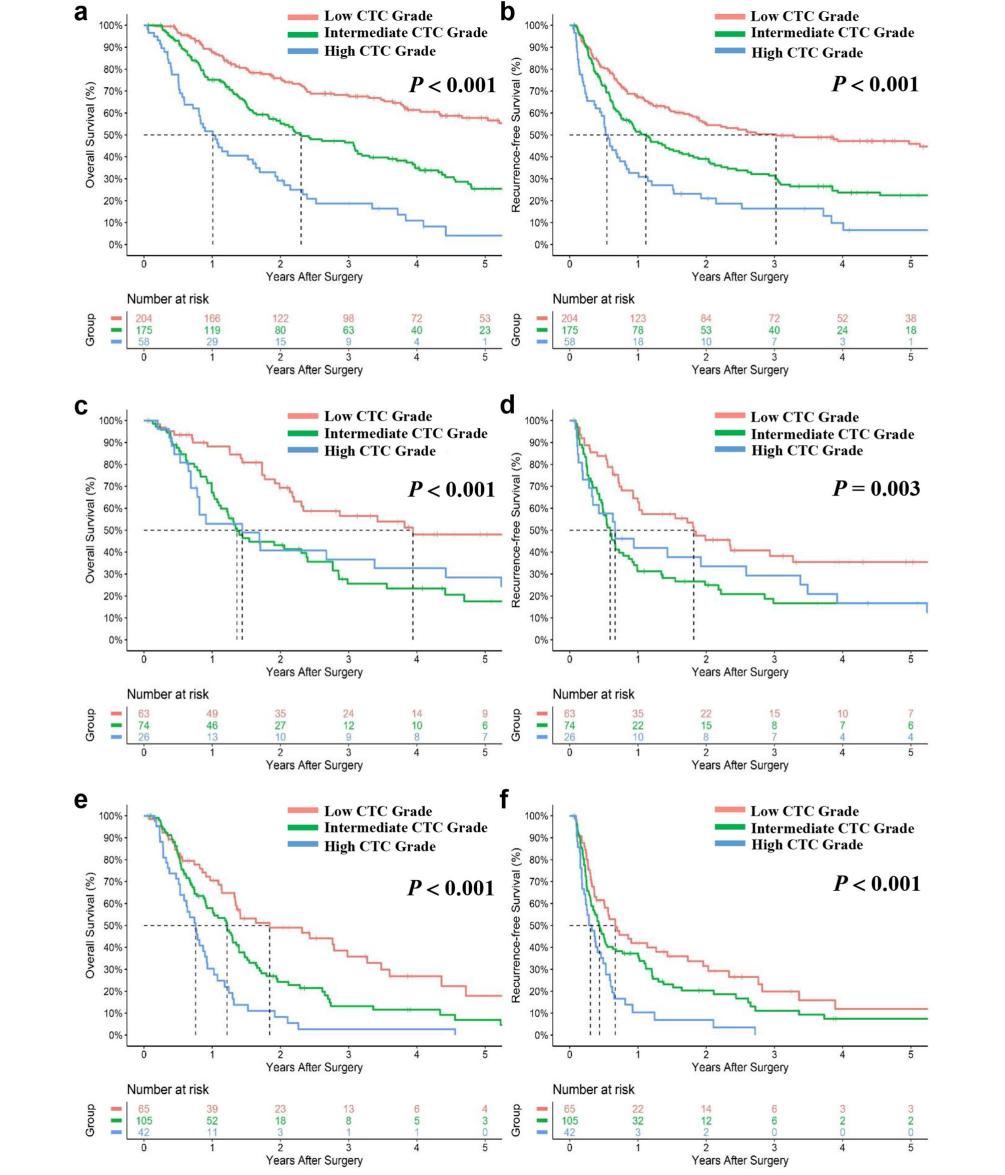
Cumulative overall survival (OS) and recurrence-free survival (RFS) curves by CTC grading for American Joint Committee on Cancer (AJCC) stages I (**a, b**), II (**c, d**), and III/IV (**e, f**) patients in the derivation cohort, respectively. CTC, combination of Tumor Burden Score and carcinoembryonic antigen

To investigate whether CTC grading system could predict outcomes across AJCC stages, subgroup analyses were performed in the derivation cohort. In the AJCC stage I group, OS and RFS worsened incrementally with higher CTC grade (for low, intermediate, and high CTC, 5 − year OS: 57.7%, 25.4%, and 4.1%, respectively; 5 − year RFS: 46.0%, 22.4%, and 6.5%, respectively; both *p* < 0.001) (Fig. 4a and b). Similarly, OS and RFS also worsened incrementally with higher CTC grade among patients with stage III/IV (for low, intermediate, and high CTC, 5 − year OS: 17.9%, 6.9%, and 0%, respectively, *p* < 0⋅001; 5 − year RFS: 11.9%, 7.4%, and 0%, respectively; *p* = 0.003) (Fig. 4e and f). Interesting, among patients with stage II, low CTC grade had better 5 − year OS and RFS compared to those with intermediate and high CTC grade (for low, intermediate and high CTC, 5 − year OS: 48.0%, 17.6%, and 28.5%, respectively, *p* < 0.001; 5 − year RFS: 35.5%, 16.6%, and 16.8%, respectively; *p* = 0.003) (Fig. 4c and d). However, patients with a high CTC grade showed better 5-year OS and RFS compared to those with an intermediate CTC grade.

## Discussion

In this study, we used a large multi-institutional database to develop and validate a simplified preoperative prognostic model, named the CTC model. This model successfully stratified long-term outcomes for patients undergoing curative-intent resection for ICC in both the derivation and validation cohorts. Our findings revealed that a high TBS grade and elevated CEA levels were independent risk factors for poor prognosis in ICC patients. These indicators demonstrated promise in predicting OS and RFS in ICC patients. When categorizing patients into low, intermediate, and high CTC groups based on varying TBS and CEA levels, we observed that patients in the high CTC group had worse OS and RFS rates compared to those in the median and low CTC groups. Consistency in these outcomes was observed in the external validation cohort. Cox regression analyses in the derivation cohort further confirmed that medium and high CTC grades were an independent risk factors for both OS (5 − year OS: low CTC, 48.9% vs. intermediate CTC, 18.7% vs. high CTC, 9.1%; *p* < 0.001) and RFS (5 − year RFS: low CTC, 38.2% vs. intermediate CTC, 17.1% vs. high CTC, 7.2%; *p* < 0.001). Importantly, the CTC grading system demonstrated moderate to well prognostic prediction capabilities in both the derivation and validation cohorts. These AUC values surpassed those of individual TBS and CEA level assessments. CTC grade system was further able to stratify prognosis within certain TNM stages (stages I and III/IV), indicating its potential as a valuable tool for estimating the prognosis of patients undergoing resection for ICC. In summary, our newly developed CTC grading system serves as a reliable prognostic predictor for ICC patients undergoing hepatectomy. This system can aid in risk stratification and treatment decision − making for ICC patients, particularly in estimating prognosis.

Relative to the potential benefits of surgical resection, risk stratification of patients is crucial for aiding in treatment decision − making and facilitating reliable discussions around prognosis with ICC patients. Our newly developed CTC grading system combines radiological TBS and serum CEA levels, both of which can be easily assessed preoperatively. Tumor markers such as CEA and CA19 − 9 have long been recognized as surrogates for tumor biology and are well − established predictors of long − term outcomes in ICC patients [28, 34, 35]. They are commonly measured in clinical practice when ICC is suspected and inform prognosis after therapies. Our data showed that elevated CEA levels predicts worse prognosis (5 − year OS: high CEA levels, 9.0% vs. low CEA levels, 36.7%; *p* < 0.001), consistent with our previous findings that preoperative elevation in serum CEA serves as a predictor of poorer outcomes [12]. In the multivariable analysis, we also observed that elevated CA19 − 9 level also serves as an adverse prognostic factor for OS. It’s important to note that we did not conduct an additional in − depth analysis of CA19 − 9 in this study. Recently, Li et al. [26] and Moazzam et al. [30] successively constructed CTC grading models based on the combination of CA19 − 9 and TBS levels, consistently demonstrated that high CTC grading is associated with poor prognosis. However, it is worth noting that they employed different approaches to determine the cutoff value for CA19 − 9 level. Li utilized the normal upper limit of 37 U/ml as the threshold, whereas Moazzam derived a cutoff value of 125 U/ml through ROC analysis. Differences in cutoff values can result in misclassification of specific patients. In our study, we employed the ROC approach to determine the cut − off value of CEA level, yielding a 5.0 U/ml. This value is in proximity to the normal upper limit of 5.0 U/ml at our center and to the cutoff value of 5.0 U/ml utilized by Sanchez et al. [7].

The concept of tumor burden, originating from the “Metro − ticket” system proposed by Mazzaferro et al. [36], highlighted a negative correlation between tumor burden and OS. Substantial studies have supported that tumor size and tumor number are important prognostic factors. Notably, tumor size and tumor number indicate the extent of the tumor in ICC, and are included in the AJCC staging systems. In the 7th edition of the AJCC staging manual, T category did not take into account tumor size [37]. Subsequently, in the revised 8th edition of the AJCC staging manual, T1 stage was further classified into T1a (isolated tumor ≤ 5 cm) and T1b (isolated tumor > 5 cm), emphasizing the effect of tumor size on outcomes [8]. These two parameters are dichotomously categorized with arbitrary cutoffs in the AJCC staging manual, which may present limitations when assessing the prognosis of patients with variable tumor sizes and nodules. The simplistic T staging system may not sufficiently offer precise information for providing personalized treatment recommendations and making decisions for patients with resectable ICC. A composite metric of tumor morphology such as TBS may be helpful in capturing total tumor burden within the liver relative to prognosis. Following this, based on the “Metro − ticket” system, Sasaki et al. [15] proposed the TBS model, which was based solely on the maximum tumor diameter and the number of lesions and represents the tumor burden in patients with CRLM. In our multivariate analysis, high TBS was a negative prognostic factor. Moreover, high TBS was associated with an inferior 5 − year RFS rate (low TBS, 32.1% vs. high TBS, 15.4%; *p* < 0.001) and 5 − year OS rate (low TBS, 40.3% vs. high TBS, 17.6%; *p* < 0.001).

Furthermore, TBS score combined with other primary tumor factors, demonstrates enhanced predictive efficacy for the prognosis of patients following ICC resection. Recently, Munir et al. [27] have demonstrated that both TBS and ALBI grade significantly influence outcomes following ICC resection. Patients with both high TBS and ALBI grade had significantly higher hazards of death compared with those who had both low TBS and ALBI grade disease (HR 2.42, 95% CI 1.57–3.73; *p* < 0.001). Ding et al. [25] developed an “AFP − TBS (ATS)” prognostic model that incorporates TBS and AFP levels at the time of initial diagnosis before surgery and at the time of recurrence to predict post − recurrence survival following the initial resection of HCC. The ATS model was negatively correlated with post − recurrence survival time and demonstrated a time − dependent AUC value of 0.70 (95% CI 0.64 − 0.75), surpassing other staging systems. Importantly, a high ATS grades significantly correlates with poorer OS and RFS outcomes, highlighting its potential clinical utility in risk stratification and treatment decision − making for ICC patients. Similar findings were observed in our study, where higher CTC grades were associated with worse RFS and OS outcomes. The CTC model, outperforming both CEA and radiographic TBS with AUC values of 0.694, 0.642, and 0.616 for OS, respectively, in pairwise comparisons (all *p* < 0.001), provides a comprehensive preoperative prognostic assessment.

It is worth noting that when analyzing subgroups across the TNM classification in the derivation cohort, patients in stage II with a high CTC grade exhibited better 5-year OS (17.6% for intermediate CTC and 28.5% for high CTC) and RFS (16.6% for intermediate CTC and 16.8% for high CTC) compared to those with an intermediate CTC grade. When interpreting this result, multiple factors may be influenced, so conclusions should be drawn with caution. Compared to high CTC grades, intermediate CTC grades are associated with higher rates of MVI (intermediate CTC: *n* = 65, 18.3% vs. high CTC: *n* = 14, 11.1%) and R1 resection (intermediate CTC: *n* = 45, 12.7% vs. high CTC: *n* = 12, 11.2%) (Table 1), which may partially influence the outcomes. MVI and R1 resection are both potential adverse prognostic factors for ICC. Additionally, incomplete pathological examination results may have underestimated the proportion of MVI. Another important factor may also be worth noting that TNM Stage II includes patients with both multiple tumors and presence of vascular invasion, implying that both multifocal tumors and vascular invasion have an equal impact on prognosis. According to our findings, the current Stage II may necessitate further subcategorization. Defects cannot belittle virtues; Our CTC grading system generally maintains good stability.

The CTC model effectively stratified OS and RFS in patients with ICC who underwent liver resection, demonstrating good predictive performance as evidenced by discrimination and calibration curves analyses. Patients in the high CTC grade group exhibited significantly lower 5-year OS and RFS rates compared to those in the low- and intermediate- CTC grade groups. Therefore, it is imperative to thoroughly discuss the risks and benefits of upfront surgery with patients who have a high CTC grade, and to ensure a more intensive follow-up postoperatively. Currently, the application of molecularly targeted agents and immunotherapies has expanded the treatment options for ICC and improved patient prognosis. A large retrospective cohort study has shown that despite typically being used for more advanced tumors, the neoadjuvant treatment is associated with significantly improved survival compared to up − front surgery for resectable ICC [38]. We hypothesized that patients with a high CTC grade might derive potential benefits from neoadjuvant therapy, and the CTC model could serve as a reference for decision-making in preoperative neoadjuvant treatment.

When interpreting the data from the current study, several limitations should be considered. Firstly, like all retrospective studies, there is the potential selection bias in choosing patients for surgical resection. Secondly, while the multi − institutional database was a strength, the absence of a control group (i.e., patients receiving alternative or non − surgical treatments) limits the generalizability to non − curative intend ICC patients. Thirdly, the exact etiology of ICC remains unclear [39, 40]; Chinese patients typically have a higher prevalence of viral hepatitis backgrounds, whereas Western patients are more commonly associated with non − alcoholic steatohepatitis. Therefore, caution should be exercised when assessing the applicability of CTC grading in Western countries, necessitating further comprehensive research and discussion on this issue. Moreover, elevated CA19 − 9 levels were also associated with adverse prognosis in the multivariable analysis. However, since this study primarily focused on the impact of TBS, CEA, and their combined CTC grading on prognosis, an extensive analysis of CA19 − 9 levels was not conducted. Therefore, future study integrating CA19 − 9 into the CTC model may enhance the performance of this model.

## Conclusion

This study demonstrated that the CTC grade performed well in stratifying patients with ICC relative to OS and RFS. It may inform preoperative discussions around prognosis and assist in identifying which patients with ICC may benefit more from neoadjuvant chemotherapy rather than up − front surgery.

### Electronic supplementary material

Below is the link to the electronic supplementary material.

Supplementary material 1

## Data Availability

No datasets were generated or analysed during the current study.
