# Supplementary material for: Tumor burden score and carcinoembryonic antigen predict outcomes in patients with intrahepatic cholangiocarcinoma following liver resection: a multi‑institutional analysis
Source: BMC Cancer. 2024 Mar 20;24:358. doi: 10.1186/s12885-024-12091-2 (PMC10953220; doi:10.1186/s12885-024-12091-2)
Supplement: Supplementary file 1 — Supplementary material 1 [file 12885_2024_12091_MOESM1_ESM.docx]

**Supplement Table 1.** Demographic and clinical characteristics of patients in the validation cohort.

| **N (%)** | **All**  **(n = 266, 100%)** | **Low CTC**  **(n = 103, 38.7%)** | **Intermediate CTC**  **(n = 125, 47.0%)** | **High CTC**  **(n = 38, 14.3%)** | ***P* value** |
| --- | --- | --- | --- | --- | --- |
| Age, years* | 54.1 ± 10.1 | 53.5 ± 9.5 | 54.3 ± 10.0 | 55.1 ± 11.6 | 0.668 |
| Gender, Male | 170 (63.9) | 69 (66.9) | 76 (60.8) | 25 (65.7) | 0.605 |
| HBV (+) | 125 (46.9) | 50 (48.5) | 57 (45.6) | 18 (47.4) | 0.905 |
| ASA score ≥ 2 | 56 (21.0) | 25 (24.2) | 23 (18.4) | 8 (21.0) | 0.557 |
| Cirrhosis | 81 (30.4) | 36 (34.9) | 33 (26.4) | 12 (31.5) | 0.372 |
| Child-Pugh grade B | 18 (6.7) | 6 (5.8) | 9 (7.2) | 3 (7.8) | 0.879 |
| Preoperative platelet counts, ×10^9^/L* | 198.3 ± 70.8 | 186.0 ± 75.7 | 200.6 ± 67.8 | 223.8 ± 60.1 | **0.017** |
| Preoperative NLR* | 2.5 (1.8 - 3.5) | 2.0 (1.7 - 2.8) | 2.8 (2.1 - 3.7) | 2.9 (1.9 - 4.3) | **< 0.001** |
| Preoperative CA 19-9, U/mL* | 36.9 (14.7 - 130.7) | 21.3 (11.5 - 44.0) | 44.1 (15.7 - 136.6) | 718.3 (46.8 -1000.0) | **< 0.001** |
| Preoperative CEA (ng/mL), n(%) |  |  |  |  | **< 0.001** |
| Low | 208 (78.2) | 103 (100.0) | 105 (84.0) | 0 (0.0) |  |
| High | 58 (21.8) | 0 (0.0) | 20 (16.0) | 38 (100.0) |  |
| Tumor Burden Score |  |  | bb |  | **< 0.001** |
| Low | 123 (46.2) | 103 (100.0) | 20 (16.0) | 0 (0.0) |  |
| High | 143 (53.8) | 0 (0.0) | 105 (84.0) | 38 (100.0) |  |
| Maximum tumor size, cm* | 6.5 ± 3.1 | 4.1 ± 1.1 | 7.8 ± 3.4 | 8.4 ± 1.7 | **< 0.001** |
| Tumor number, multiple | 42 (15.7) | 14 (13.5) | 21 (16.8) | 7 (18.4) | 0.716 |
| Tumor differentiation |  |  |  |  | 0.773 |
| Well | 16 (6.0) | 5 (4.8) | 8 (6.4) | 3 (7.8) |  |
| Poor to moderate | 250 (94.0) | 98 (95.2) | 117 (93.6) | 35 (92.2) |  |
| Type of resection, Major | 62 (23.3) | 18 (17.4) | 31 (24.8) | 13 (34.2) | 0.098 |
| Macrovascular invasion | 32 (12.0) | 7 (6.7) | 16 (12.8) | 9 (23.6) | **0.022** |
| Microvascular invasion | 28 (10.5) | 8 (7.7) | 14 (11.2) | 6 (15.7) | 0.366 |
| Resection margin status |  |  |  |  | 0.281 |
| R0 | 235 (88.4) | 87 (84.5) | 113 (90.4) | 35 (92.2) |  |
| R1 | 31 (11.6) | 16 (15.5) | 12 (9.6) | 3 (7.8) |  |
| Postoperative adjuvant therapy | 81 (30.4) | 31 (30.0) | 41 (32.8) | 9 (23.6) | 0.562 |
| AJCC staging system^8th^ |  |  |  |  | **< 0.001** |
| I | 128 (48.1) | 66 (64.0) | 54 (43.2) | 8 (21.0) |  |
| II | 89 (33.4) | 27 (26.3) | 46 (36.8) | 16 (42.2) |  |
| III | 49 (18.5) | 10 (9.7) | 25 (20.0) | 14 (36.8) |  |
| Postoperative 30-day complication | 99 (37.2) | 39 (37.8) | 43 (34.4) | 17 (44.7) | 0.506 |
| Minor morbidity | 78 (29.4) | 32 (31.1) | 33 (26.4) | 13 (34.2) | 0.576 |
| Major morbidity | 21 (7.8) | 7 (6.7) | 10 (8.0) | 4 (10.5) | 0.765 |

ASA, American Society of Anesthesiologists; AJCC, American Joint Committee on Cancer; CA 19-9, carbohydrate antigen 19-9; CEA, carcinoembryonic antigen; CTC, combination of Tumor Burden Score and CEA; HBV, hepatitis B virus; NLR, neutrophil-to-lymphocyte ratio.

* Values are mean ± standard deviation or median (interquartile range) unless otherwise indicated.

**Supplement Table 2.** Univariable and multivariable Cox-regression analysis for overall survival (OS) of patients in the validation cohort

| **Variables** | **HR comparison** | **UV HR (95% CI)** | **UV *P* value** | **MV HR (95% CI)** | **MV *P* value** |
| --- | --- | --- | --- | --- | --- |
| Age | > 60 *vs.* ≤ 60 years | 1.19 (0.83 - 1.71) | 0.323 |  |  |
| Gender | Male *vs.* Female | 1.08 (0.76 - 1.53) | 0.651 |  |  |
| HBV (+) | HBV *vs.* non-HBV | 0.84 (0.60 - 1.18) | 0.337 |  |  |
| ASA score | ≥ 2 *vs.* ＜ 2 | 0.81 (0.53 - 1.23) | 0.329 |  |  |
| Cirrhosis | Yes *vs.* No | 1.02 (0.83 - 1.16) | 0.166 |  |  |
| Child-Pugh grade | B *vs.* A | 1.12 (0.59 - 2.14) | 0.711 |  |  |
| Preoperative platelet counts | < 100 *vs.* ≥ 100 × 10^9^/L | 0.63 (0.31 -1.29) | 0.211 |  |  |
| Preoperative NLR | > 4 *vs.* ≤ 4 | 1.64 (1.09 - 2.46) | **0.016** | NS | 0.653 |
| Preoperative CA 19-9 | > 37 *vs.* ≤ 37 U/mL | 1.77 (1.26 - 2.47) | **< 0.001** | 1.55 (1.05 - 2.07) | **0.023** |
| Tumor differentiation | Poor or moderate *vs.* Well | 2.02 (0.82 - 4.93) | 0.122 |  |  |
| Type of resection | Major *vs.* Minor | 1.41 (0.97 - 2.04) | 0.068 |  |  |
| Macrovascular invasion | Yes *vs.* No | 1.40 (1.25 - 1.79) | **0.016** | 1.30 (1.15 - 1.72) | **0.036** |
| Microvascular invasion | Yes *vs.* No | 2.03 (1.29 - 3.21) | **0.002** | 1.96 (1.22 - 3.23 | **0.009** |
| Resection margin status | R1 *vs.* R0 | 1.63 (1.28 - 1.97) | **0.012** | NS | 0.661 |
| Postoperative adjuvant therapy | Yes *vs.* No | 0.97 (0.68 - 1.38) | 0.890 |  |  |
| AJCC staging system^8th^ | III/IV *vs.* I/II | 3.16 (2.17 - 4.58) | **< 0.001** | 2.58 (1.75 - 3.79) | **< 0.001** |
| CTC grade | Intermediate *vs.* Low | 2.32 (1.56 - 3.47) | **< 0.001** | 1.87 (1.22 - 2.85) | **0.005** |
|  | High *vs.* Low | 4.15 (2.55 - 6.77) | **< 0.001** | 2.70 (1.58 - 4.65) | **< 0.001** |

ASA, American Society of Anesthesiologists; AJCC, American Joint Committee on Cancer; CA 19-9, carbohydrate antigen 19-9; CEA, carcinoembryonic antigen; CTC, combination of Tumor Burden Score and CEA; CI, confidence interval; HBV, hepatitis B virus; HR, hazard ratio; MV, multivariable; NLR, neutrophil-to-lymphocyte ratio; NS, not significant; UV, univariable.

**Supplement Table 3.** Univariable and multivariable Cox-regression analysis for recurrence-free survival (RFS) of patients in the validation cohort

| **Variables** | **HR comparison** | **UV HR (95% CI)** | **UV *P* value** | **MV HR (95% CI)** | **MV *P* value** |
| --- | --- | --- | --- | --- | --- |
| Age | > 60 *vs.* ≤ 60 years | 0.91 (0.65 - 1.27) | 0.609 |  |  |
| Gender | Male *vs.* Female | 0.93 (0.68 - 1.25) | 0.627 |  |  |
| HBV (+) | HBV *vs.* non-HBV | 1.00 (0.75 - 1.35) | 0.952 |  |  |
| ASA score | ≥ 2 *vs.* ＜ 2 | 1.11 (0.77 - 1.58) | 0.562 |  |  |
| Cirrhosis | Yes *vs.* No | 1.00 (0.73 - 1.38) | 0.955 |  |  |
| Child-Pugh grade | B *vs.* A | 1.14 (0.66 - 1.97) | 0.629 |  |  |
| Preoperative platelet counts | < 100 *vs.* ≥ 100 × 10^9^/L | 0.78 (0.43 -1.41) | 0.411 |  |  |
| Preoperative NLR | > 4 *vs.* ≤ 4 | 1.35 (0.92 - 1.96) | 0.116 |  |  |
| Preoperative CA 19-9 | > 37 *vs.* ≤ 37 U/mL | 1.77 (1.31 - 2.38) | **< 0.001** | 1.55 (1.12 - 2.16) | **0.003** |
| Tumor differentiation | Poor or moderate *vs.* Well | 1.34 (0.68 - 2.63) | 0.378 |  |  |
| Type of resection | Major *vs.* Minor | 1.24 (0.88 - 1.73) | 0.206 |  |  |
| Macrovascular invasion | Yes *vs.* No | 1.54 (1.11 - 2.01) | **0.002** | 1.48 (1.06 - 1.98) | **0.033** |
| Microvascular invasion | Yes *vs.* No | 1.97 (1.28 - 3.05) | **0.002** | 1.70 (1.05 - 2.71) | **0.018** |
| Resection margin status | R1 *vs.* R0 | 1.43 (0.98 - 1.75) | 0.077 |  |  |
| Postoperative adjuvant therapy | Yes *vs.* No | 1.21 (0.88 - 1.64) | 0.227 |  |  |
| AJCC staging system^8th^ | III/IV *vs.* I/II | 3.06 (2.17 - 4.31) | **< 0.001** | 2.55 (1.79 - 3.62) | **< 0.001** |
| CTC grade | Intermediate *vs.* Low | 2.06 (1.47 - 2.90) | **< 0.001** | 1.59 (1.14 - 2.34) | **0.010** |
|  | High *vs.* Low | 2.78 (2.78 - 4.35) | **< 0.001** | 1.97 (1.20 - 3.18) | **0.003** |

ASA, American Society of Anesthesiologists; AJCC, American Joint Committee on Cancer; CA 19-9, carbohydrate antigen 19-9; CEA, carcinoembryonic antigen; CTC, combination of Tumor Burden Score and CEA; CI, confidence interval; HBV, hepatitis B virus; HR, hazard ratio; MV, multivariable; NLR, neutrophil-to-lymphocyte ratio; NS, not significant; UV, univariable.

**Supplement Figure 1.**


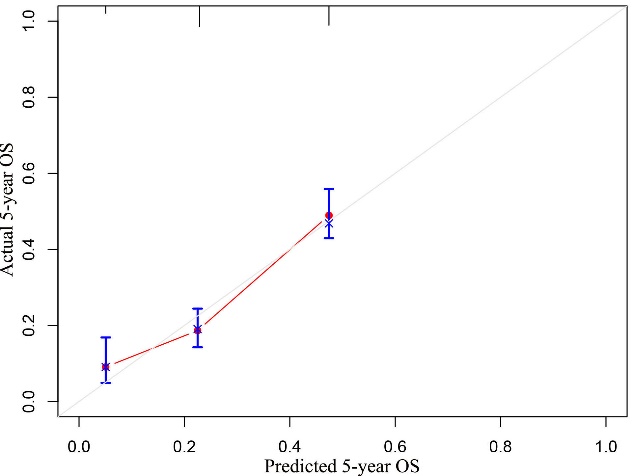

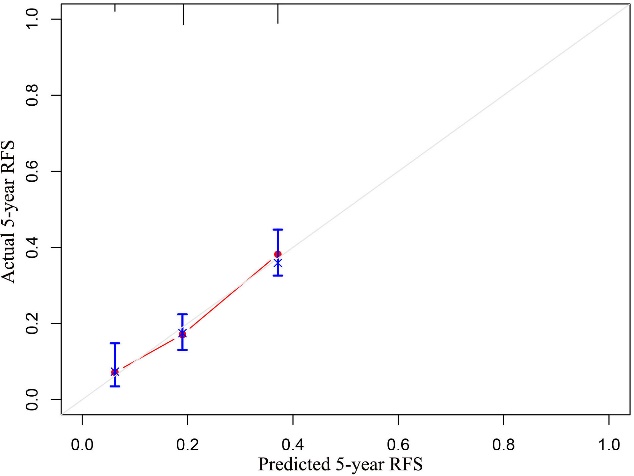


**a**

**b**

**H-L test *P* = 0.629**

**H-L test *P* = 0.559**


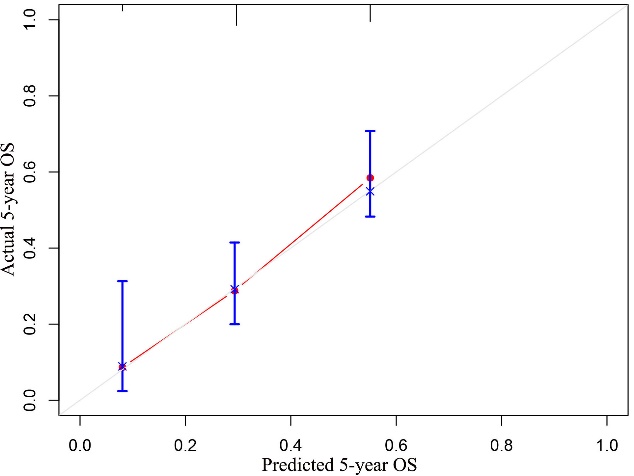

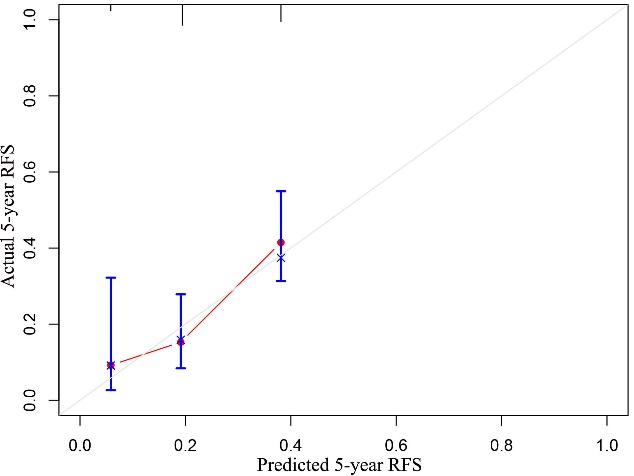


**H-L test *P* = 0.249**

**H-L test *P* = 0.939**

**d**

**c**

**Supplement Figure 1.** Calibration curves for CTC grading to predict overall survival (OS) and recurrence-free survival (RFS) in derivation (**A, B**) and validation (**C, D**) cohorts. H-L, Hosmer-Lemeshow.
